# Supplementary material for: Dorsal root ganglion macrophages contribute to both the initiation and persistence of neuropathic pain
Source: Nat Commun. 2020 Jan 14;11:264. doi: 10.1038/s41467-019-13839-2 (PMC6959328; doi:10.1038/s41467-019-13839-2)
Supplement: Supplementary file 1 — Supplementary Information [file 41467_2019_13839_MOESM1_ESM.pdf]

SUPPLEMENTARY INFORMATION

Dorsal root ganglion macrophages contribute to both the initiation and persistence of neuropathic pain

Yu et al.

Figure S1

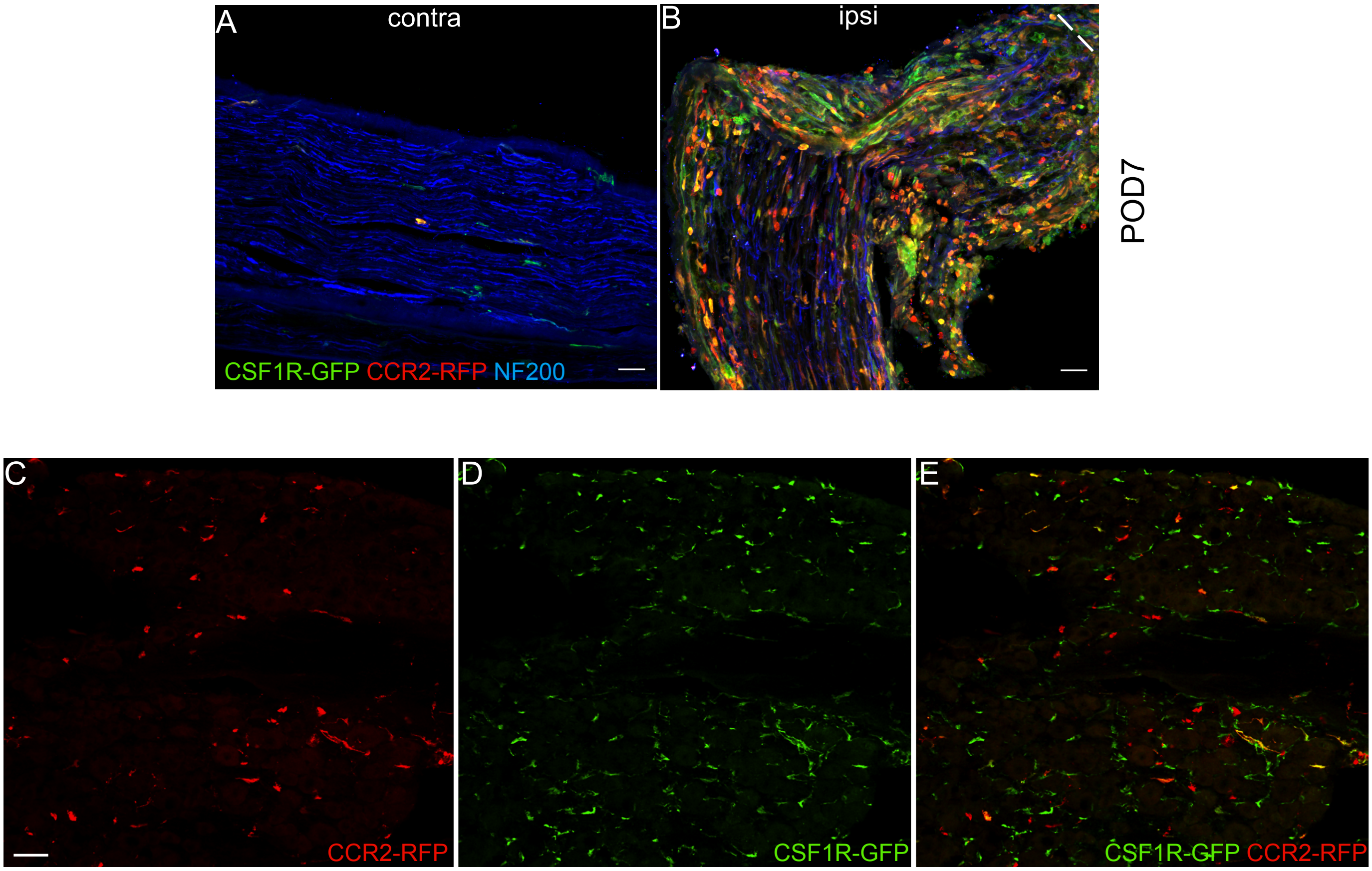
A
contra
B
ipsi
POD7
CSF1R-GFP CCR2-RFP NF200
C
D
E
CCR2-RFP
CSF1R-GFP
CSF1R-GFP CCR2-RFP

Supplementary Figure 1: Macrophage distribution in peripheral nerve and DRG. (**A-B**) Immunostaining of macrophages co-expressing CSF1R-GFP (green) and CCR2-RFP (red) in the sciatic nerve of a CSF1R-GFP$^{+/-}$CCR2-RFP$^{+/-}$ mouse on POD7; contralateral (A) and ipsilateral (B) to the injury. Dashed line in upper right corner of B denotes ligature site; NF200 (blue) marks myelinated axons. Scale bar: 10 µm in A-B. (**C-E**) Immunostaining of macrophages co-expressing CSF1R-GFP (green) and CCR2-RFP (red) in the L4/L5 DRG of uninjured CSF1R-GFP$^{+/-}$CCR2-RFP$^{+/-}$ mice. Scale bar: 50µm in C-E.

Figure S2

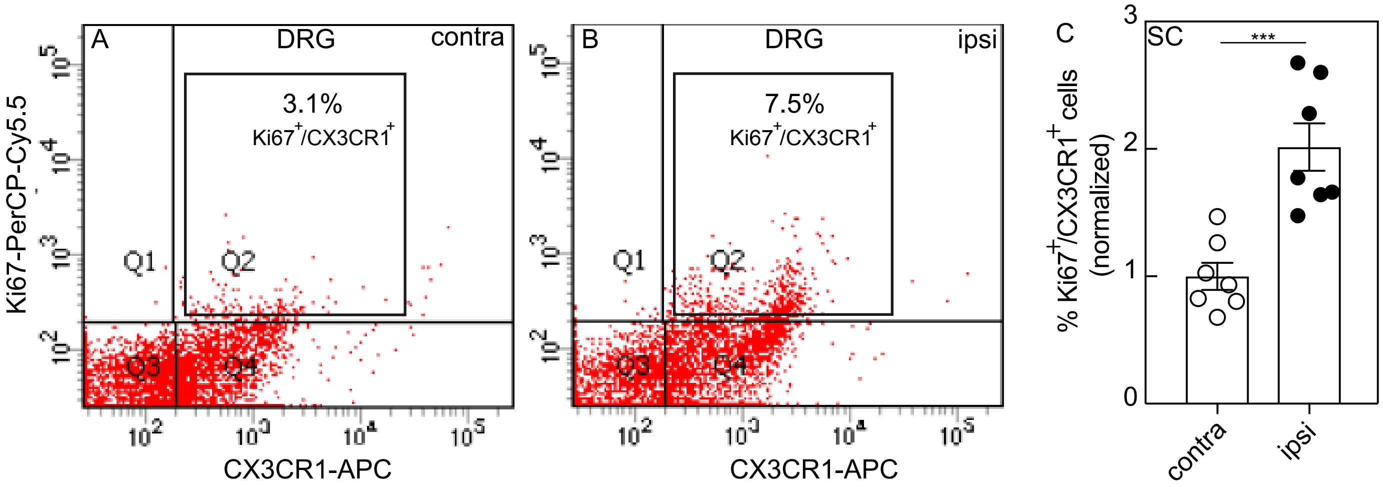

A
DRG
contra
3.1%
Ki67+/CX3CR1+
Q1
Q2
Q3
Q4
Ki67-PerCP-Cy5.5
CX3CR1-APC
B
DRG
ipsi
7.5%
Ki67+/CX3CR1+
Q1
Q2
Q3
Q4
CX3CR1-APC
C
SC
***
% Ki67+/CX3CR1+ cells (normalized)
contra
ipsi

Supplementary Figure 2. Profilerating CX3CR1$^+$ cells in the DRG and lumbar spinal cord of wild-type mice after SNI. Dissociated cells were immunostained with CX3CR1-APC and Ki67-PerCp-Cy5.5 antibodies. (**A**-**B**) Representative FACS analysis of Ki67 expression in CX3CR1$^+$ macrophages in the contralateral (A) and ipsilateral (B) L4/L5 DRG 4 days after nerve injury (POD4). The percentage of gated cells are noted in the upper right quadrant. (**C**) FACS analysis of Ki67 expression in CX3CR1$^+$ lumbar spinal cord (SC) microglia on POD4 (n=7 per group). Data presented as mean ± S.E.M. Student's *t*-test in C. ***P<0.001.

Figure S3

A

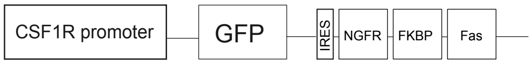
CSF1R promoter
GFP
IRES
NGFR
FKBP
Fas

B

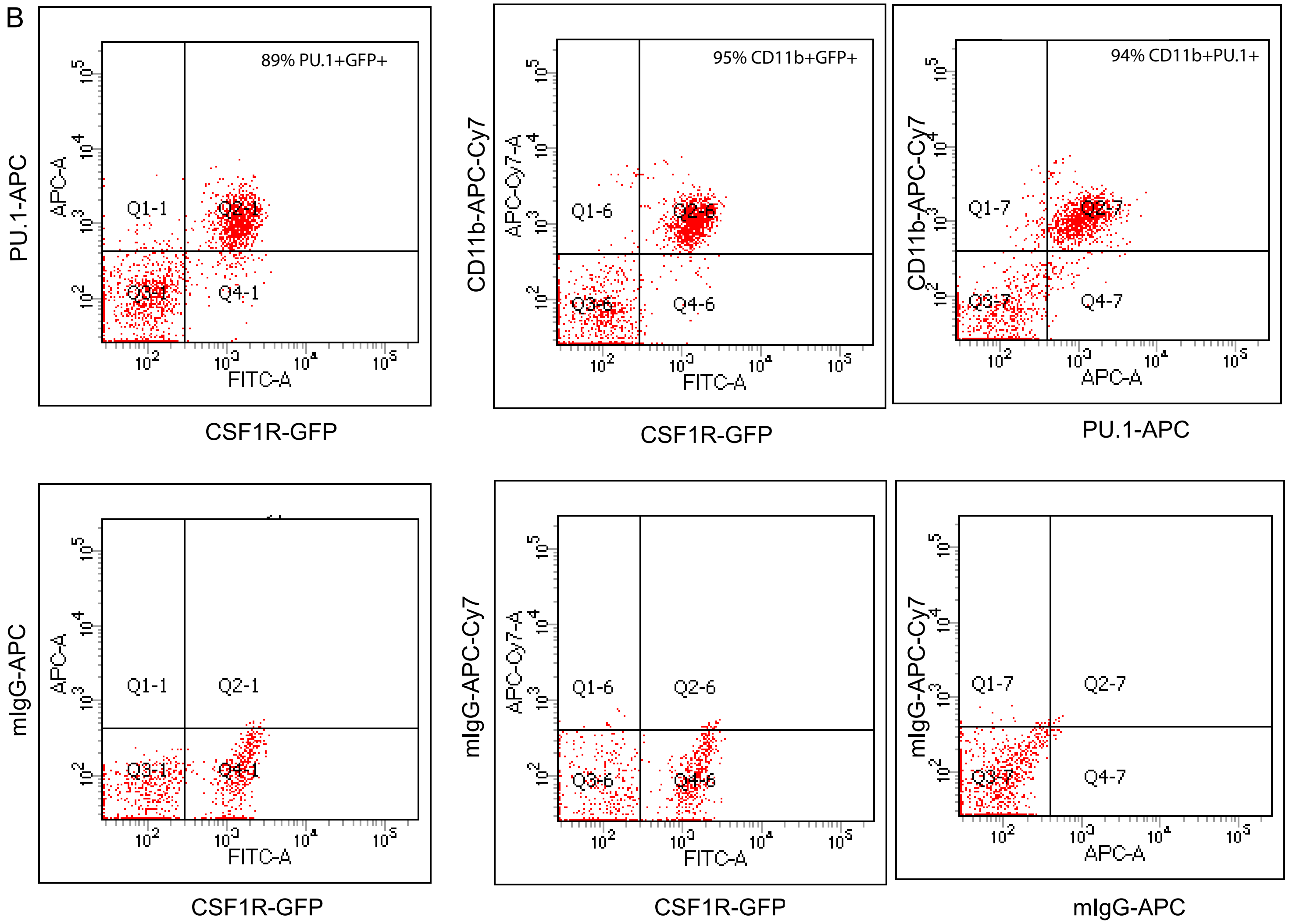
89% PU.1+GFP+
PU.1-APC
APC-A
FITC-A
Q1-1
Q2-1
Q3-1
Q4-1
CSF1R-GFP
95% CD11b+GFP+
CD11b-APC-Cy7
APC-Cy7-A
Q1-6
Q2-6
Q3-6
Q4-6
CSF1R-GFP
94% CD11b+PU.1+
CD11b-APC-Cy7
Q1-7
Q2-7
Q3-7
Q4-7
APC-A
PU.1-APC
mIgG-APC
CSF1R-GFP
mIgG-APC-Cy7
CSF1R-GFP
mIgG-APC-Cy7
mIgG-APC

Supplementary Figure 3. Characterization of transgenic CSF1R-GFP^{+} spinal cord microglia. (**A**) Schematic of the transgene in MAFIA mice. (**B**) FACS analysis of dissociated spinal cord microglia from naïve MAFIA mice immunostained for PU.1-APC and CD11b-APC-Cy7 (specific monocytic cell markers) antibodies. The threshold for positivity was based on the background fluorescence in cells incubated with mIgG-APC or mIgG-APC-Cy7 isotype control antibody (lower panels). The percentage of gated cells are noted in the upper right quadrant.

Figure S4

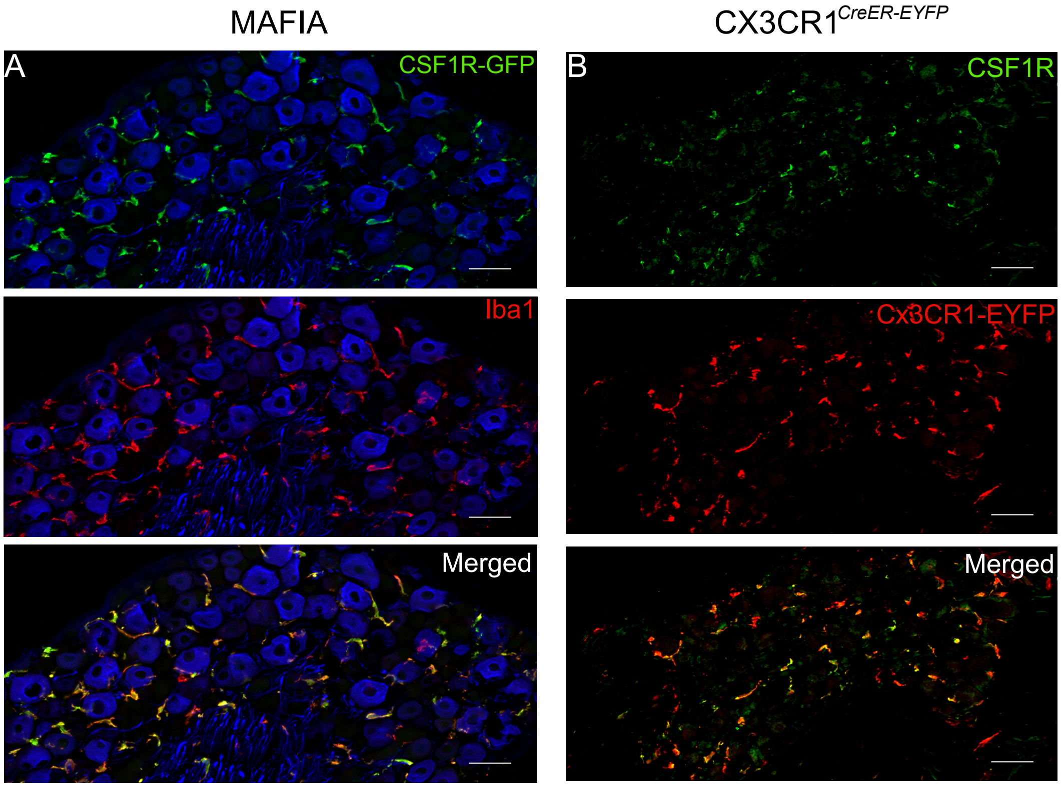
MAFIA
$CX3CR1^{CreER-EYFP}$
A
CSF1R-GFP
Iba1
Merged
B
CSF1R
Cx3CR1-EYFP
Merged

Supplementary Figure 4. Characterization of DRG macrophages. (**A**) Immunoreactive macrophage markers (CSF1R-GFP, green; Iba-1, red), and a neuronal marker (NF200, blue) in the DRG of MAFIA mice. (**B**) Immunoreactive CSF1R (green) and CX3CR1-EYFP (red) in the DRG of CX3CR1$^{CreER-EYFP}$ mice. Scale bar: 50 μm.

Figure S5

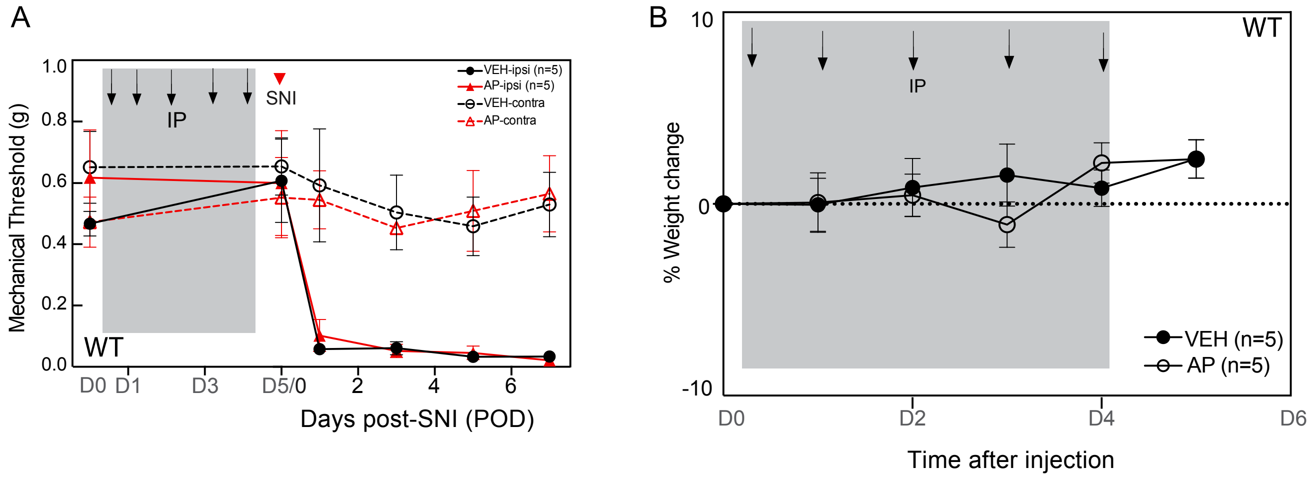
A
Mechanical Threshold (g)
1.0
0.8
0.6
0.4
0.2
0.0
IP
SNI
VEH-ipsi (n=5)
AP-ipsi (n=5)
VEH-contra
AP-contra
WT
D0 D1 D3 D5/0 2 4 6
Days post-SNI (POD)
B
% Weight change
10
0
-10
IP
WT
VEH (n=5)
AP (n=5)
D0 D2 D4 D6
Time after injection

Supplementary Figure 5. AP20187 does not affect pre- or post- nerve injury mechanical thresholds or weight in WT mice. (**A**) Mechanical thresholds before (D0 to D5) and for several days after SNI (POD1-7) following high dose systemic AP (10 mg kg^{-1} x 5 days) or VEH in WT mice (n=5 per group). (**B**) Body weight change in the treated WT mice (n=5 per group). Grey shading indicates injection days. Data presented as mean ± S.E.M. Two-way repeated measures ANOVA with Sidak's correction.

Figure S6

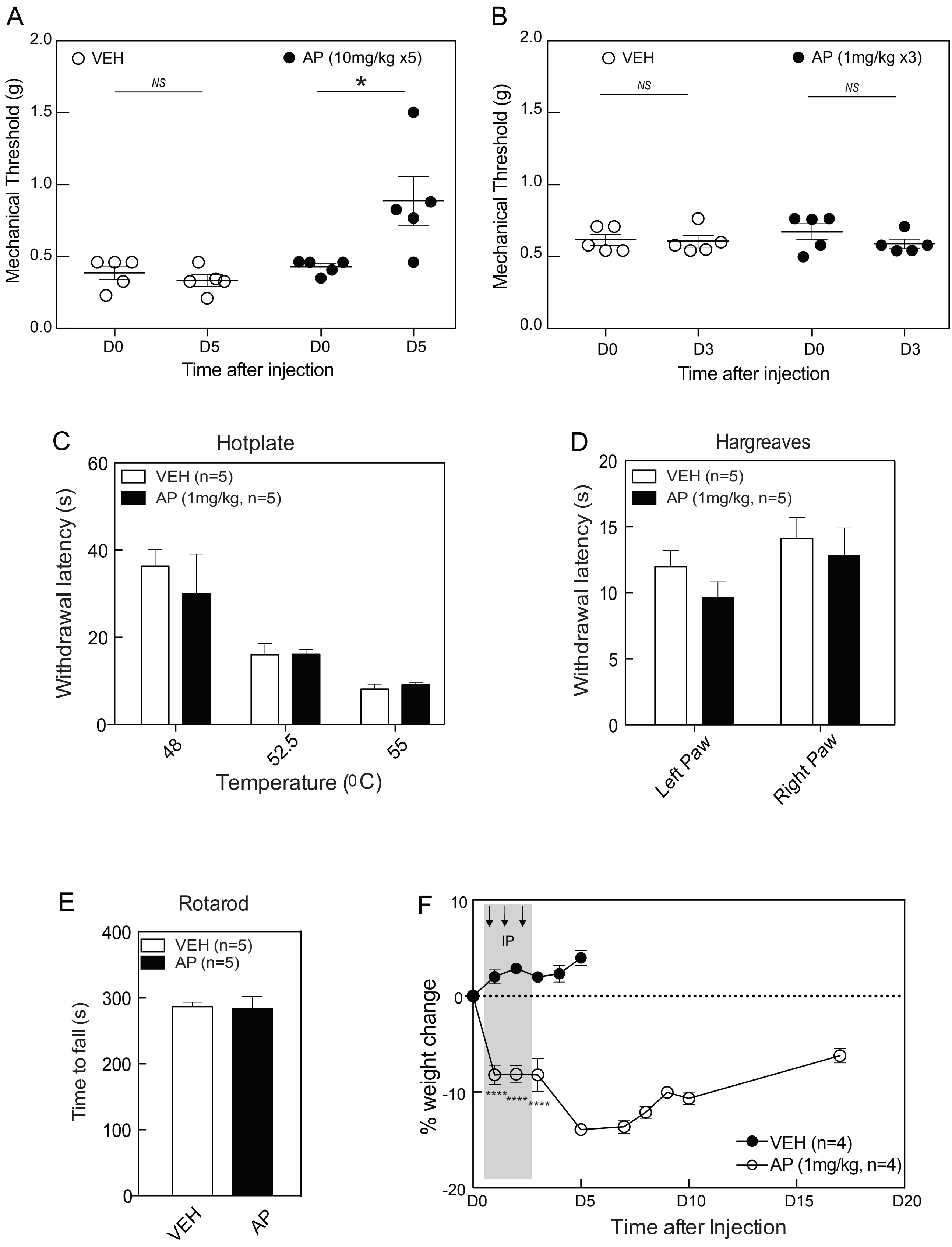
A
○ VEH
● AP (10mg/kg x5)
NS
*
Mechanical Threshold (g)
2.0
1.5
1.0
0.5
0.0
D0
D5
D0
D5
Time after injection
B
○ VEH
● AP (1mg/kg x3)
NS
NS
Mechanical Threshold (g)
D0
D3
D0
D3
Time after injection
C
Hotplate
VEH (n=5)
AP (1mg/kg, n=5)
Withdrawal latency (s)
60
40
20
0
48
52.5
55
Temperature (0C)
D
Hargreaves
VEH (n=5)
AP (1mg/kg, n=5)
Withdrawal latency (s)
20
15
10
5
0
Left Paw
Right Paw
E
Rotarod
VEH (n=5)
AP (n=5)
Time to fall (s)
400
300
200
100
0
VEH
AP
F
IP
% weight change
10
0
-10
-20
****
****
****
VEH (n=4)
AP (1mg/kg, n=4)
D0
D5
D10
D15
D20
Time after Injection

Supplementary Figure 6: Effect of systemic AP on baseline mechanical and thermal (heat) thresholds and motor coordination in MAFIA mice. (**A**) Mechanical threshold before and after 5 days of high dose systemic AP (10 mg kg^{-1}) or VEH in MAFIA mice (n=5 per group). (**B-E**) Mechanical threshold (B), latency to respond in the hot plate test (C), withdrawal latency in the Hargreaves test (D) and motor coordination in the rotarod test (E) after 3 days low dose systemic AP (1.0 mg kg^{-1}) or VEH (n=5 per group). **(F)** Weight change after low dose systemic AP or VEH (n=4 per group). Grey shading indicates injection days. Data presented as mean ± S.E.M. Student's *t*-test in A, B and E. Two-way ANOVA with Sidak's correction in C, D and F. *P<0.05, *NS*, nonsignificant compared to control.

Figure S7

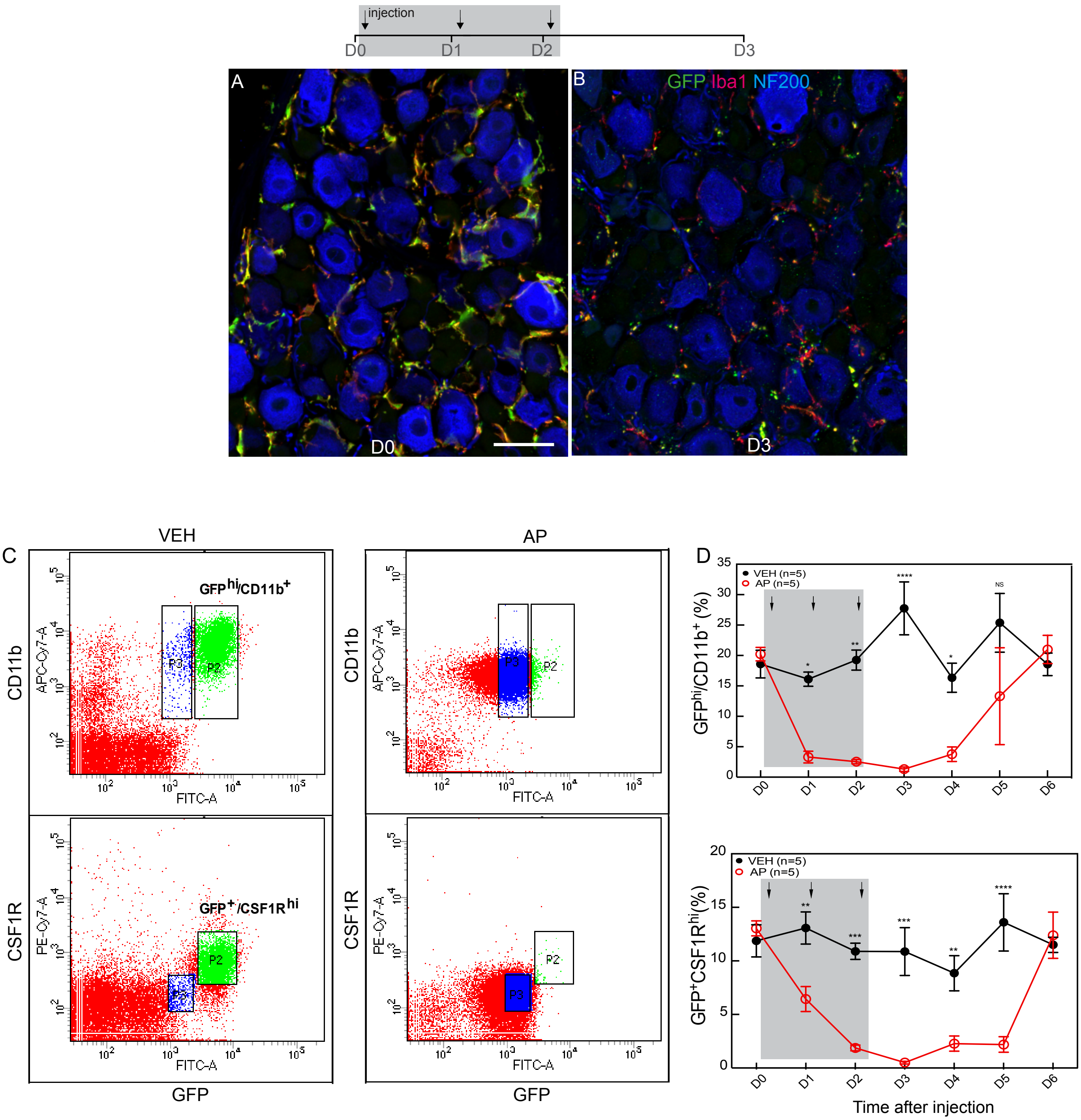

injection
D0
D1
D2
D3
A
B
GFP Iba1 NF200
D0
D3
C
VEH
AP
CD11b
CSF1R
APC-Cy7-A
PE-Cy7-A
FITC-A
GFP
GFPhi/CD11b+
GFP+/CSF1Rhi
P2
P3
D
VEH (n=5)
AP (n=5)
GFPhi/CD11b+ (%)
GFP+CSF1Rhi(%)
NS
D0
D1
D2
D3
D4
D5
D6
Time after injection

Supplementary Figure 7. Systemic AP depletes both DRG macrophages and blood monocytes.

(**A**) Colocalization of $CSF1R^+$ (green) and $Iba1^+$ (red) macrophages in the DRG, before (D0) and 3 days (D3) after systemic AP (3 x 1.0 mg kg^{-1}). NF200 (blue) marks large diameter neurons. Scale bar: 50 μm. (**C**-**D**) FACS analysis of blood monocytes after 3 daily systemic AP (1.0 mg kg^{-1}) or VEH injections. **(C)** Representative blood monocyte FACS 1 day after the 3rd injection (VEH: left; AP: right) with the gated monocyte populations indicated: Green (P2): $GFP^{hi}CD11b^+$ and GFP^+CSF1R^{hi}; Blue (P3): $GFP^{lo}CD11b^+$ and GFP^+CSF1R^{lo}. (**D**) Percentage of circulating monocytes before and after systemic AP or VEH (n=5 mice per group). Grey shading indicates injection days. Data presented as mean ± S.E.M. Two-way repeated measures ANOVA with Sidak's correction. *P<0.05, **P<0.01, ***P<0.001, ****P<0.0001, *NS*, nonsignificant compared to control.

Figure S8

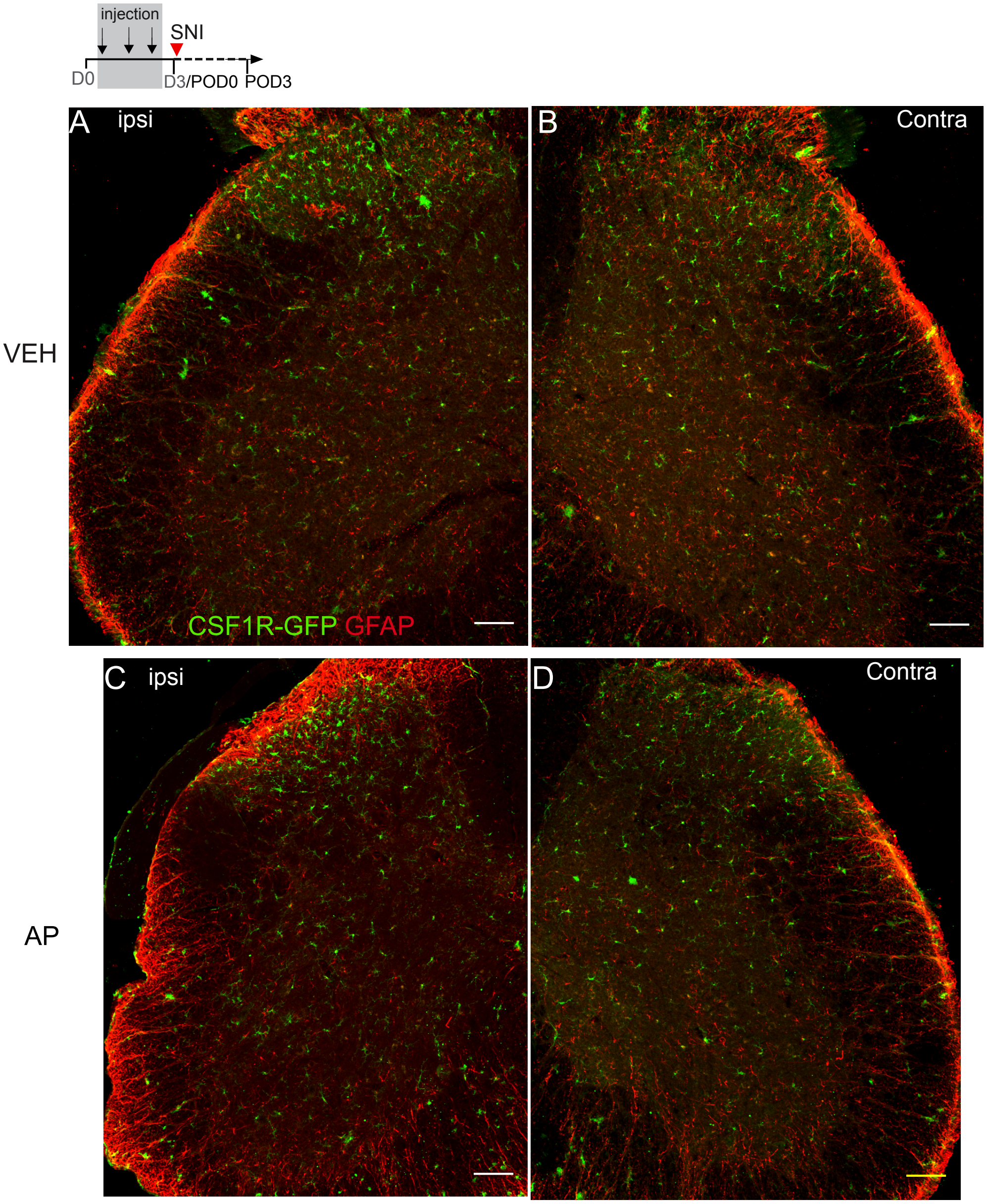

injection
SNI
D0
D3/POD0
POD3
A
ipsi
B
Contra
VEH
CSF1R-GFP GFAP
C
ipsi
D
Contra
AP

Supplementary Figure 8: Systemic AP treatment does not influence spinal cord astrocytes. (**A**-**D**) Immunoreactive microglia (CSF1R-GFP, green) and astrocytes (GFAP, red) in the ipsilateral (A, C) and contralateral (B, D) lumbar spinal cord 4 days after SNI (POD4) following systemic AP (1 mg kg^{-1} x 3 days, C-D) or VEH (A-B) in MAFIA mice. Grey shading indicates injection days. Scale bar: 15 µm.

Figure S9

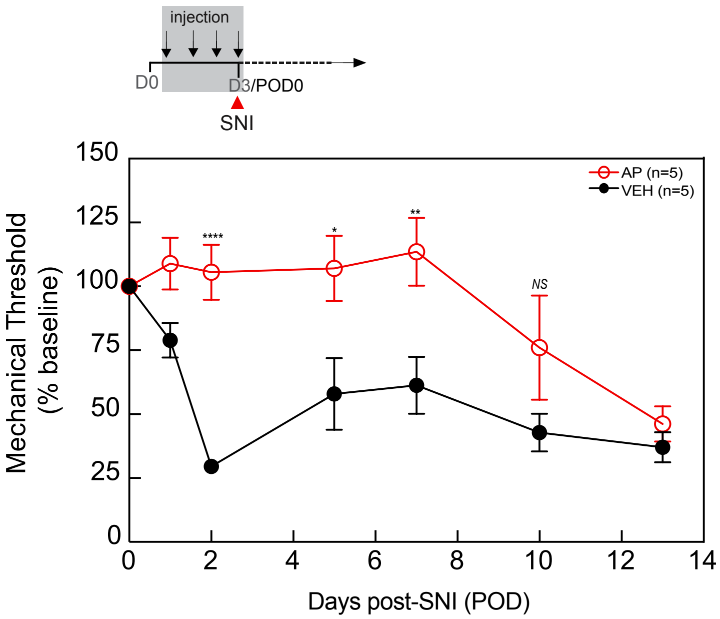

injection
D0
D3/POD0
SNI
Mechanical Threshold (% baseline)
150
125
100
75
50
25
0
AP (n=5)
VEH (n=5)
****
*
**
NS
0
2
4
6
8
10
12
14
Days post-SNI (POD)

Supplementary Figure 9. Extension of AP treatment to cover the day of nerve injury did not further delay development of the mechanical hypersensitivity in MAFIA mice. Mechanical thresholds after systemic AP (1 mg kg^{-1}) or VEH (n=5 per group) before and after SNI. Grey shading indicates injection days. Data presented as mean ± S.E.M. One-way ANOVA with Tukey's correction**.** *P<0.05, **P<0.01, ****P<0.0001, *NS*, nonsignificant compared to control.

Figure S10

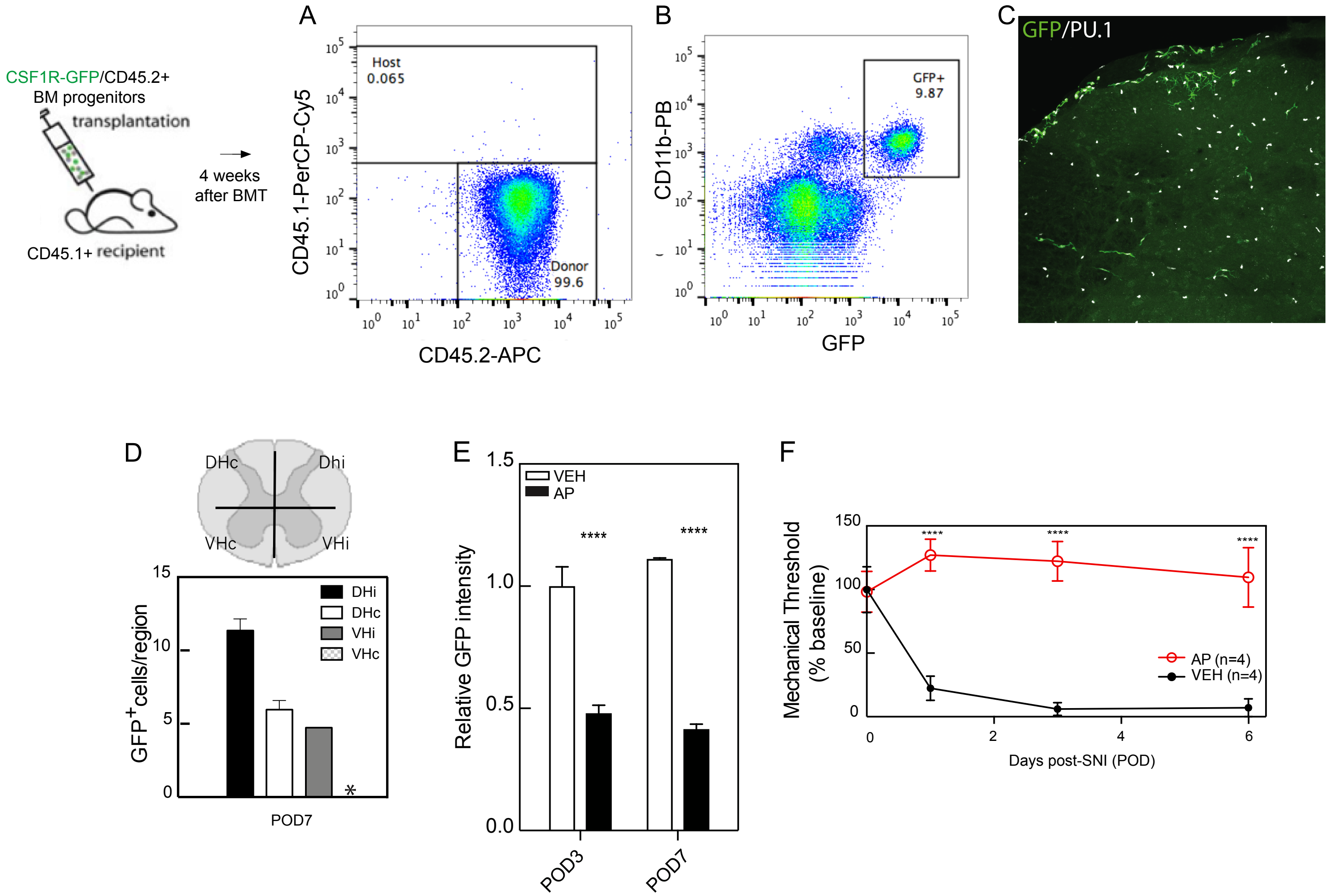

CSF1R-GFP/CD45.2+
BM progenitors
transplantation
4 weeks
after BMT
CD45.1+ recipient
A
Host
0.065
Donor
99.6
CD45.1-PerCP-Cy5
CD45.2-APC
B
GFP+
9.87
CD11b-PB
GFP
C
GFP/PU.1
D
DHc
Dhi
VHc
VHi
DHi
DHc
VHi
VHc
GFP+cells/region
POD7
E
VEH
AP
****
Relative GFP intensity
POD3
POD7
F
Mechanical Threshold
(% baseline)
AP (n=4)
VEH (n=4)
Days post-SNI (POD)

Supplementary Figure 10: Transplantation of hematopoietic bone marrow (BM) progenitor cells from MAFIA mice into irradiated WT mice does not influence resident spinal cord microglia. Lineage-depleted BM progenitor donor cells ($CD45.2^+$) isolated from MAFIA mice (CSF1R-GFP) were transplanted into lethally irradiated $CD45.1^+$ WT recipient mice. (**A-B**) Blood was drawn from the tail vein at 4 weeks after BM transplantation and analyzed by FACS for myeloid cell engraftment with an anti-45.1-PerCP-Cy5 (recipient cell marker) and anti-45.2-APC (donor cell marker) antibodies (A) or with an anti-CD11b-PB antibody (B). (**C**) Immunostaining for PU.1 (white, marker of all microglia) and GFP (green, a marker of donor cells) in the ipsilateral lumbar dorsal horn of BM transplanted mice 7 days after SNI (POD7). (**D**) Quantitation of GFP^+ donor microglial cells in the four regions of lumbar spinal cord on POD7 (5-8 sections per mouse; n=4 mice). DHi, ipsilateral dorsal horn; DHc, contralateral dorsal horn; VHi, ipsilateral ventral horn; VHc, contralateral ventral horn. *, no GFP^+ cells detected. **(E**) GFP intensity in the ipsilateral DRG on POD3 and POD7 in VEH or systemic AP-treated transplanted mice (n=3). (**F**) Mechanical threshold of AP and VEH-treated transplanted mice at different times after SNI (n=4 per group). Data presented as mean ± SEM. 2-way ANOVA with Sidak's correction in F and G. ****P<0.0001.

Figure S11

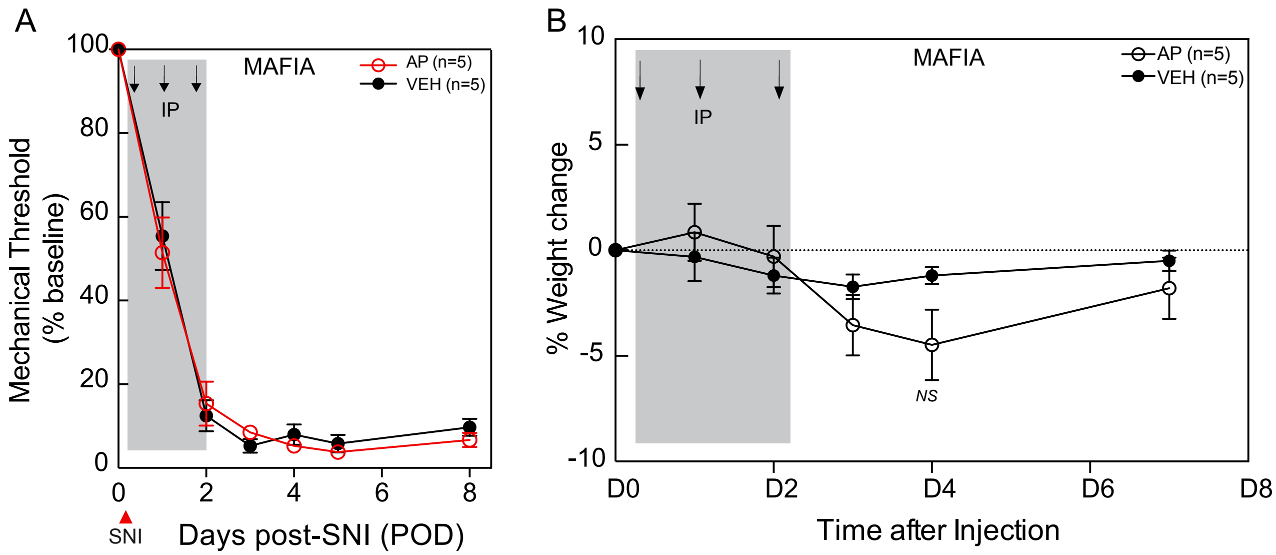
A
100
80
60
40
20
0
Mechanical Threshold
(% baseline)
MAFIA
IP
AP (n=5)
VEH (n=5)
0
2
4
6
8
SNI
Days post-SNI (POD)
B
10
5
0
-5
-10
% Weight change
MAFIA
IP
AP (n=5)
VEH (n=5)
NS
D0
D2
D4
D6
D8
Time after Injection

Supplementary Figure 11. Lack of effect of low-dose systemic AP in MAFIA mice. (**A-B**) Neither post-SNI mechanical threshold (A) nor weight (B) was altered by systemic low-dose AP (0.8 μg x 3) or VEH (n=5 per group). Grey shading indicates treatment days. Data presented as mean ± SEM; Two-way repeated measures ANOVA with Sidak's correction. *NS*, nonsignificant compared to control.

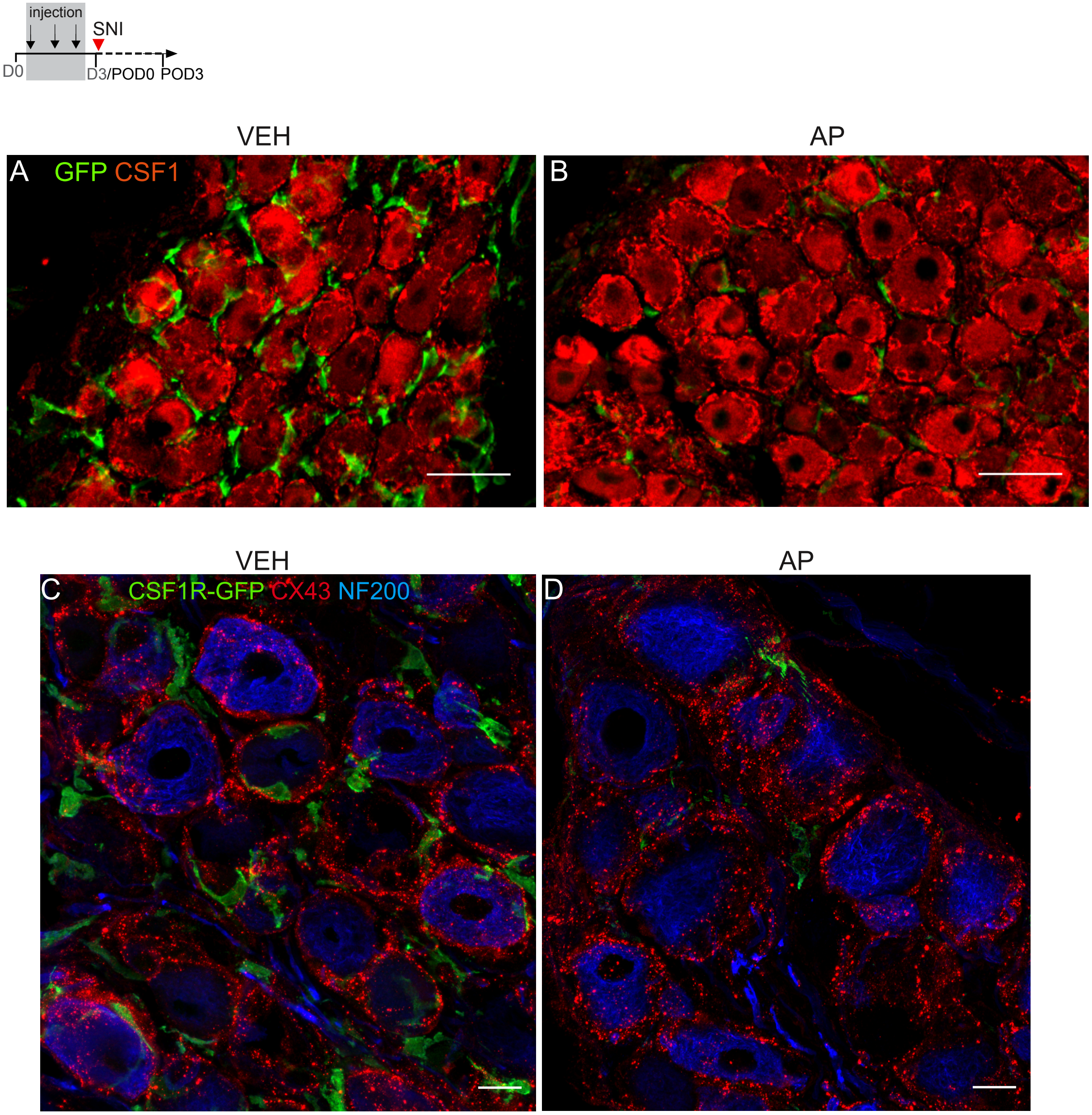

injection
SNI
D0
D3/POD0
POD3
VEH
AP
A
GFP CSF1
B
VEH
AP
C
CSF1R-GFP CX43 NF200
D

Supplementary Figure 12. **Systemic AP treatment did not prevent nerve injury induction of neuronal CSF1 and did not alter satellite cells after SNI.** Systemic injection of MAFIA mice with 1.0 mg kg^{-1} AP or VEH for 3 days, followed by SNI. **(A-D)** Immunostaining for CSF1 (red, A-B) or Connexin 43, a satellite cell marker (red, C-D) in the axotomized DRG on POD3. CSF1R-GFP (green) labels macrophages; NF200 (blue) labels neuronal cell bodies (C-D). Grey shading denotes injection days. Scale bars: 50 μm in A-B; 15 μm in C-D.

Figure S13

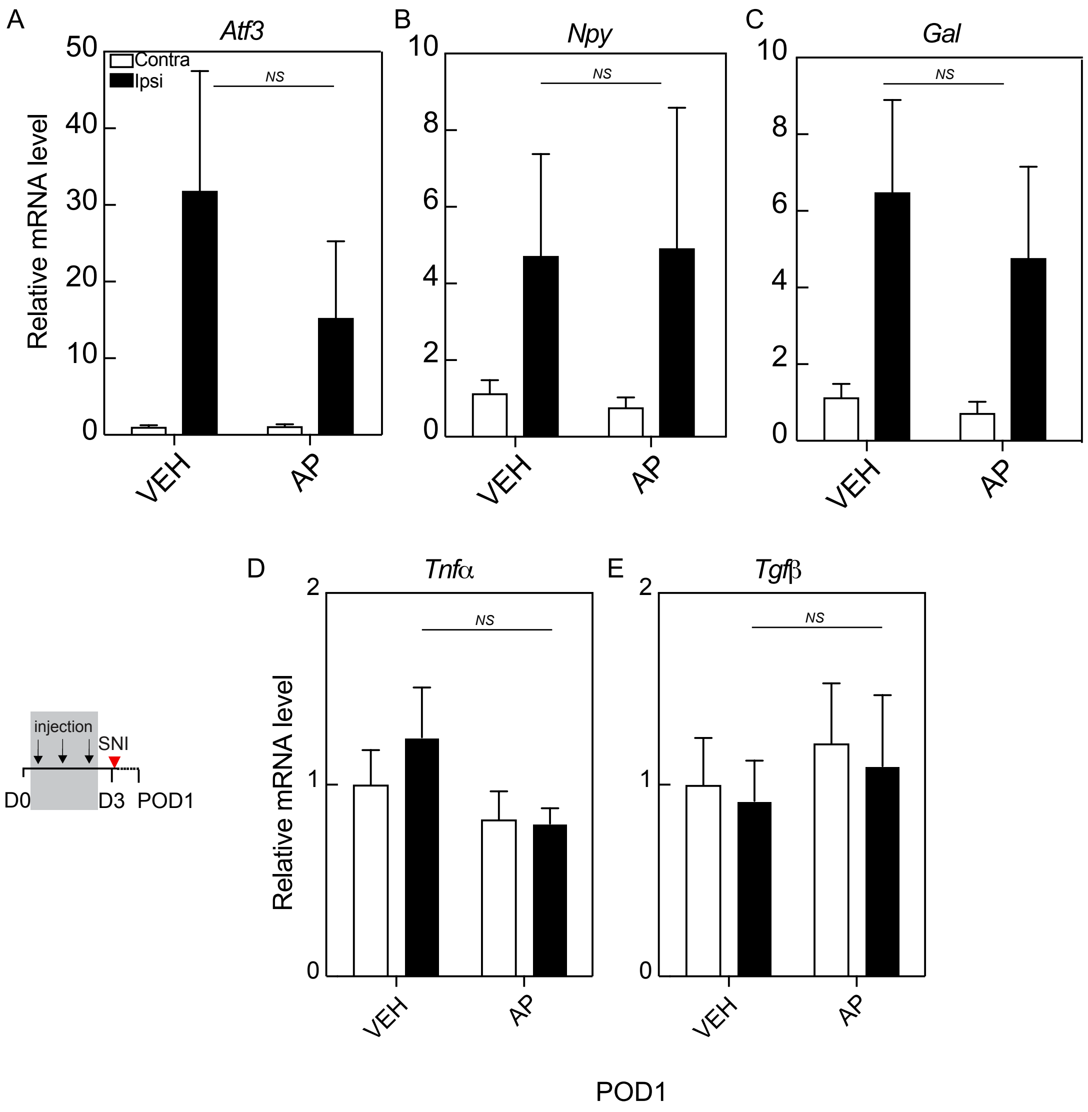

A
Atf3
Contra
Ipsi
NS
Relative mRNA level
50
40
30
20
10
0
VEH
AP
B
Npy
NS
10
8
6
4
2
0
VEH
AP
C
Gal
NS
10
8
6
4
2
0
VEH
AP
injection
SNI
D0
D3
POD1
D
Tnfα
NS
Relative mRNA level
2
1
0
VEH
AP
E
Tgfβ
NS
2
1
0
VEH
AP
POD1

Supplementary Figure 13. Nerve injury-induced genes in the DRG not altered by systemic AP treatment. (**A-E**) Systemic injection of MAFIA mice with 1.0 mg kg^{-1} AP or VEH for 3 days, followed by SNI. qPCR of gene expression in L4/5 DRG 1 day after SNI (POD1): (A) activating transcription factor 3 (*Atf3*), (B) neuropeptide Y (*Npy*), (C) galanin (*Gal*), (D) tumor necrosis factor α (*Tnf*α) and (E) transforming growth factor β (*Tgf*β). n=3 per group. Grey shading denotes injection days. Data presented as mean ± SEM. Two-way ANOVA; *NS*, nonsignificant compared to VEH.

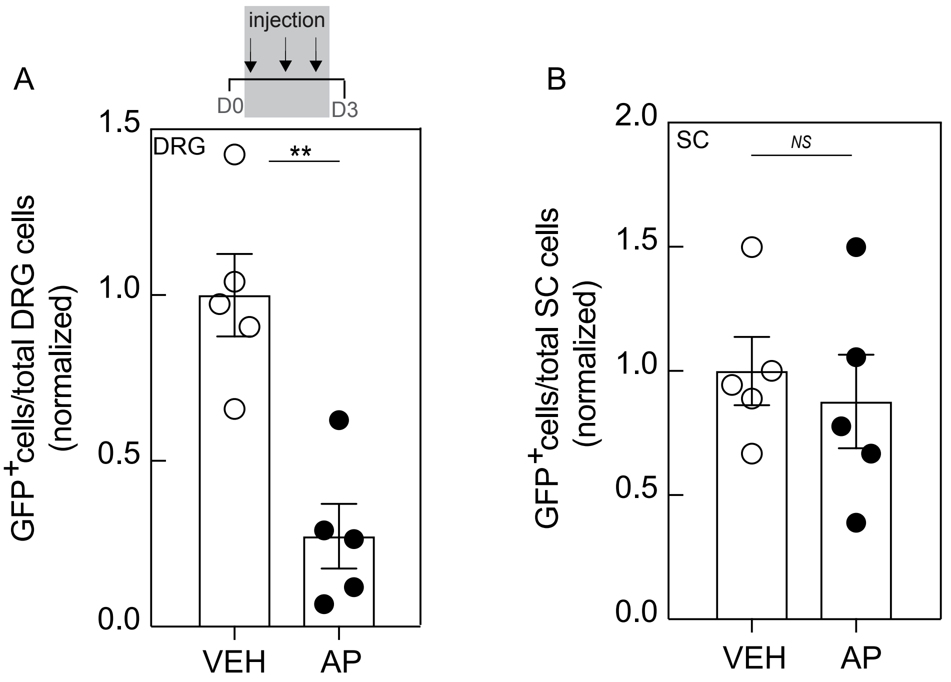
A
injection
D0
D3
1.5
1.0
0.5
0.0
DRG
**
GFP+cells/total DRG cells (normalized)
VEH
AP
B
2.0
1.5
1.0
0.5
0.0
SC
NS
GFP+cells/total SC cells (normalized)
VEH
AP

Supplementary Figure 14. Systemic AP treatment depletes DRG macrophages but does not affect spinal cord (SC) microglia in female MAFIA mice. (**A**) FACS analysis of CSF1R-GFP$^+$ female DRG macrophages 1 day after the 3rd AP (1.0 mg kg^{-1}) or VEH injection (n=5 per group). (**B**) FACS analysis of CSF1R-GFP$^+$ female spinal cord microglia after 3-day AP or VEH injections (n=5 per group). Grey shading denotes injection days. Data presented as mean ± SEM. Student's *t*-test in A and B. $^{**}P<0.01$, *NS*, nonsignificant compared to control.
