# Supplementary material for: Dorsal root ganglion macrophages contribute to both the initiation and persistence of neuropathic pain
Source: Nat Commun. 2020 Jan 14;11:264. doi: 10.1038/s41467-019-13839-2 (PMC6959328; doi:10.1038/s41467-019-13839-2)
Supplement: Supplementary file 3 — Reporting Summary [file 41467_2019_13839_MOESM3_ESM.pdf]

nature research

Corresponding author(s): DBPR NCOMMS-19-15154B

Last updated by author(s): Nov 7, 2019

Reporting Summary

Nature Research wishes to improve the reproducibility of the work that we publish. This form provides structure for consistency and transparency in reporting. For further information on Nature Research policies, see Authors & Referees and the Editorial Policy Checklist.

Statistics

For all statistical analyses, confirm that the following items are present in the figure legend, table legend, main text, or Methods section.

n/a	Confirmed	
☐	☒	The exact sample size (*n*) for each experimental group/condition, given as a discrete number and unit of measurement
☐	☒	A statement on whether measurements were taken from distinct samples or whether the same sample was measured repeatedly
☐	☒	The statistical test(s) used AND whether they are one- or two-sided<br>*Only common tests should be described solely by name; describe more complex techniques in the Methods section.*
☐	☒	A description of all covariates tested
☐	☒	A description of any assumptions or corrections, such as tests of normality and adjustment for multiple comparisons
☐	☒	A full description of the statistical parameters including central tendency (e.g. means) or other basic estimates (e.g. regression coefficient) AND variation (e.g. standard deviation) or associated estimates of uncertainty (e.g. confidence intervals)
☐	☒	For null hypothesis testing, the test statistic (e.g. *F*, *t*, *r*) with confidence intervals, effect sizes, degrees of freedom and *P* value noted<br>*Give P values as exact values whenever suitable.*
☒	☐	For Bayesian analysis, information on the choice of priors and Markov chain Monte Carlo settings
☒	☐	For hierarchical and complex designs, identification of the appropriate level for tests and full reporting of outcomes
☐	☒	Estimates of effect sizes (e.g. Cohen's *d*, Pearson's *r*), indicating how they were calculated

Our web collection on statistics for biologists contains articles on many of the points above.

Software and code

Policy information about availability of computer code

Data collection	N/A
Data analysis	The GraphPad PRISM software for statistical analysis. Image J for image processing.

For manuscripts utilizing custom algorithms or software that are central to the research but not yet described in published literature, software must be made available to editors/reviewers. We strongly encourage code deposition in a community repository (e.g. GitHub). See the Nature Research guidelines for submitting code & software for further information.

Data

Policy information about availability of data

All manuscripts must include a data availability statement. This statement should provide the following information, where applicable:

- Accession codes, unique identifiers, or web links for publicly available datasets
- A list of figures that have associated raw data
- A description of any restrictions on data availability

All data included in the manuscript is available from the corresponding author on reasonable request.

Field-specific reporting

Please select the one below that is the best fit for your research. If you are not sure, read the appropriate sections before making your selection.

☒ Life sciences ☐ Behavioural & social sciences ☐ Ecological, evolutionary & environmental sciences

For a reference copy of the document with all sections, see nature.com/documents/nr-reporting-summary-flat.pdf

Life sciences study design

All studies must disclose on these points even when the disclosure is negative.

Sample size	Sample sizes for behavioral, cellular biological and histochemical studies were based on previous experiments from our laboratory and others, which had been demonstrated to be capable of detecting significant changes.
Data exclusions	No data exclusions
Replication	Experiments were performed with sufficient animals per group to demonstrate statistical significance.
Randomization	The animals were randomly assigned to the treatment group and control group. For behavioral experiments, animals were initially placed into one cage and allowed to free run for a few minutes. Next each animal was randomly picked up and placed into a separate cylinder before the behavior test.
Blinding	Experimenters were blinded to the animals for behavioral study.

Reporting for specific materials, systems and methods

We require information from authors about some types of materials, experimental systems and methods used in many studies. Here, indicate whether each material, system or method listed is relevant to your study. If you are not sure if a list item applies to your research, read the appropriate section before selecting a response.

Materials & experimental systems

n/a	Involved in the study	
☐	☒	Antibodies
☒	☐	Eukaryotic cell lines
☒	☐	Palaeontology
☐	☒	Animals and other organisms
☒	☐	Human research participants
☒	☐	Clinical data

Methods

n/a	Involved in the study	
☒	☐	ChIP-seq
☐	☒	Flow cytometry
☒	☐	MRI-based neuroimaging

Antibodies

Antibodies used	Antibodies used for histochemistry: chicken anti-GFP (1:2000, Abcam #ab13970), rabbit anti-CSF1R (1:15000, Millipore #06-174), rabbit anti-Iba1 (1:2000, Wako #019-19741), rabbit anti-PU.1 (1:500, Cell Signaling #2266), mouse anti-NF200 (1:1000, Sigma #N5389), rabbit anti-dsRed (1:500; Clontech #632393), rabbit anti-Connexin 43 (1:2000, Sigma #C6219), rabbit anti-GFAP (1:20000, DAKO #Z0334), goat anti-CSF1 (1:500, R&D #AF416).<br>The following antibodies used for FACS: anti-CD115-PE cy7 (1:1000, eBioscience #25115282), CD11b-APC-cy7 (1:2000. Biolegend #101226), PU.1 (1:1000, Cell Signaling #2266), chicken anti-GFP antibody (1:1000, Abcam #ab13970), anti-CX3CR1-APC (1:2000, Biolegend #149008) or anti-Ki67-PerCP-efluro710 (1:2000, Invitrogen, #66-5698-82)
Validation	For validation, the following methods were used: 1) use of isotype controls for FACS analysis, 2) results from previous publications from our lab, 3) manufacturer provided validation on the same species.

Animals and other organisms

Policy information about studies involving animals; ARRIVE guidelines recommended for reporting animal research

Laboratory animals	Both male and female adult mice (4 to 12 weeks old) were used in all experiments. Wild-type C57BL/6 mice, MAFIA (CSF1R-EGFP-NGFR/FKBP1A/TNFRSF6) transgenic mice (Stock #005070), CCL2 knockout mice (Stock #004434) and CCR2-RFP+/+ knock-in mice (Stock #017586) were obtained from the Jackson Laboratory. We also studied CX3CR1CreER-EYFP mice originally generated by Wen-Biao Gan at New York University. We crossed homozygous MAFIA mice with homozygous CCR2-RFP+/+ mice to generate CSF1R-GFP+/-CCR2-RFP+/- mice.
Wild animals	N/A
Field-collected samples	N/A
Ethics oversight	All animal experiments were approved by the Institutional Animal Care and Use Committee at UCSF and were conducted in accordance with the NIH Guide for the Care and Use of Laboratory animals

Note that full information on the approval of the study protocol must also be provided in the manuscript.

Flow Cytometry

Plots

Confirm that:

☒ The axis labels state the marker and fluorochrome used (e.g. CD4-FITC).

☒ The axis scales are clearly visible. Include numbers along axes only for bottom left plot of group (a 'group' is an analysis of identical markers).

☒ All plots are contour plots with outliers or pseudocolor plots.

☒ A numerical value for number of cells or percentage (with statistics) is provided.

Methodology

Sample preparation	Peripheral blood was drawn from a retro-orbital vein in isoflurane anesthetized mice using heparinized micropipettes (ThermoFisher Scientific #22362566). Red blood cells were lysed first before immunostaining. Fresh isolated spinal cord tissue was digested with collagenase, dissociated and filtered through cell strainers (70 µm). Microglia were further enriched using a myelin removal beads protocol (Miltenyi Biotech #130-096-733). Alternatively, freshly dissected lumbar cord or L4/L5 DRG was first homogenized in cold calcium and magnesium-free Hanks' balanced salt solution. After filtering through a cell strainer (70 µm), the cell homogenate was mixed with Percoll (Sigma) for myelin removal and enrichment. Dissociated cells were briefly fixed in 4% paraformaldehyde before intracellular immunostaining.
Instrument	BD FACS CantoII
Software	BD FACSDiva
Cell population abundance	Cells were not sorted in this study.
Gating strategy	Preliminary FSC/SSC gating was determined based on our experiences and further confirmed by viability assay (PI or 7AAD) and reporter (EGFP) analysis. Wild-type mouse cells without GFP expression were used to gate GFP+ or RFP+ cells. To determine "positive" cell population stained with an antibody, appropriate isotype control was used for gating. In addition, reporter gene expression (CSF1RGFP) was used initially to confirm the monocytic cell marker costaining for macrophages and microglial cells.

☒ Tick this box to confirm that a figure exemplifying the gating strategy is provided in the Supplementary Information.
